# Stratification at the health district level for targeting malaria control interventions in Mali

**DOI:** 10.1038/s41598-022-11974-3

**Published:** 2022-05-18

**Authors:** Mady Cissoko, Mahamadou Magassa, Vincent Sanogo, Abdoulaye Ouologuem, Lansana Sangaré, Modibo Diarra, Cedric Stephane Bationo, Mathias Dolo, Mamadou Djoulde Bah, Sidy Doumbia, Mamadou B. Coulibaly, Diahara Traoré, Boubacar Sidibé, Jordi landier, Idrissa Cissé, Moussa Sacko, Jean Gaudart, Issaka Sagara

**Affiliations:** 1 Malaria Research and Training Center (MRTC), FMOS-FAPH, Mali-NIAID-ICER, Université des Sciences, des Techniques et des Technologies de Bamako, 1805, Bamako, Mali; 2 Direction Régionale de la Santé de Tombouctou, 59, Tombouctou, Mali; 3 IRD, INSERM, Aix Marseille Univ, ISSPAM, SESSTIM, UMR1252, Faculty of Medicine, 13005 Marseille, France; 4 Programme National de Lutte Contre le Paludisme (PNLP), 233, Bamako, Mali; 5 Institut National de Santé Publique (INSP), 1771, Bamako, Mali; 6 Laboratoire de Biologie Moléculaire Appliquée (LBMA), E3206, Bamako, Mali; 7 President’s Malaria Initiative (PMI) VectorLink Project City Niger, 612, Bamako, Mali; 8 Organisation Mondiale de la Santé au Mali, 99, Bamako, Mali; 9 Aix Marseille Univ, APHM, INSERM, IRD, SESSTIM, ISSPAM, UMR1252, Hop Timone, BioSTIC, Biostatistic & ICT, Faculty of Medicine, 13005 Marseille, France

**Keywords:** Epidemiology, Malaria

## Abstract

Malaria is the leading cause of morbidity and mortality in Mali. Between 2017 and 2020, the number of cases increased in the country, with 2,884,827 confirmed cases and 1454 reported deaths in 2020. We performed a malaria risk stratification at the health district level in Mali with a view to proposing targeted control interventions. Data on confirmed malaria cases were obtained from the District Health Information Software 2, data on malaria prevalence and mortality in children aged 6–59 months from the 2018 Demographic and Health Survey, entomological data from Malian research institutions working on malaria in the sentinel sites of the National Malaria Control Program (NMCP), and environmental data from the National Aeronautics and Space Administration. A stratification of malaria risk was performed. Targeted malaria control interventions were selected based on spatial heterogeneity of malaria incidence, malaria prevalence in children, vector resistance distribution, health facility usage, child mortality, and seasonality of transmission. These interventions were discussed with the NMCP and the different funding partners. In 2017–2019, median incidence across the 75 health districts was 129.34 cases per 1000 person-years (standard deviation = 86.48). Risk stratification identified 12 health districts in very low transmission areas, 19 in low transmission areas, 20 in moderate transmission areas, and 24 in high transmission areas. Low health facility usage and increased vector resistance were observed in high transmission areas. Eight intervention combinations were selected for implementation. Our work provides an updated risk stratification using advanced statistical methods to inform the targeting of malaria control interventions in Mali. This stratification can serve as a template for continuous malaria risk stratifications in Mali and other countries.

## Introduction

Malaria is the leading cause of morbidity and mortality among children in Mali. The number of cases increased in the country over the 2017–2020 period. In 2020, the surveillance system reported 2,884,827 confirmed cases (including 871,265 severe cases) out of 4,252,213 people tested^1^. That same year, health facilities reported 1454 deaths due to malaria, and the disease was the most frequent reason for health care visits (accounting for 36% of consultations)^1^. As these data indicate, reducing the malaria burden would considerably lower child mortality in Mali, which was still high at 101 per 1000 live births in 2018^2^.

The main parasites responsible for malaria in Mali are *Plasmodium falciparum* (more than 85% of responsible parasites), *Plasmodium malariae* (10–15%), and *Plasmodium ovale* (1%)^3^, though *Plasmodium vivax* has also been observed and documented^4,5^. Malaria is endemic in most localities, and its transmission increases with the rainy season. The most common vector species are *Anopheles gambiae s.l.* (*Anopheles gambiae s.s.*, *Anopheles coluzzii*, and *Anopheles arabiensis*) and *Anopheles funestus*^6^, which have been found to develop resistance to the insecticides used^7,8^. The main mechanisms of resistance to available insecticides are the Alpha esterizes, Beta esterase, Kdr, and Ace- 1 genes^9^.

In accordance with World Health Organization (WHO) technical guidelines^10^, Mali has implemented a malaria control strategy based on case management and prevention. This strategy is coordinated at different levels by the National Malaria Control Program (NMCP). Malaria case management involves early diagnostic by rapid diagnostic test (RDT) and prompt treatment. The first-line treatments used in Mali are artemether-lumefantrine combination therapy for simple malaria and artesunate injection for severe malaria^1^. Prevention interventions consist of vector control using long-lasting insecticidal nets (LLINs) and indoor residual spraying (IRS), seasonal chemoprevention (SMC), and intermittent preventive treatment in pregnancy (IPTp). In Mali, LLINs are distributed every 3 years in all health districts via mass campaigns and are routinely provided to pregnant women and children under 1 year of age. Indoor residual spraying was carried out in 8 health districts in 2017 and in 2 health districts in 2020. Indeed, the main vector species, *An. gambiae*, has developed resistance to older insecticides, and the new insecticides (pirimiphos-methyl^11,12^ and clothianidin^13^) are too costly to be used on a large scale. Seasonal malaria chemoprevention was first implemented in Mali in 2012 and has been performed in all health districts during periods of high malaria transmission since 2016. It consists in the administration of sulfadoxine-pyrimethamine (SP) and amodiaquine (AQ) to children under 5 years of age once per month from July to October (1 SP tablet and 1 AQ tablet on day 1, 1 AQ tablet on day 2, and 1 AQ tablet on day 3 of each of month). Lastly, IPTp, namely the administration of SP to pregnant women from the 13th week of pregnancy, has been implemented in all health districts since 2000^1^.

In malaria-endemic countries, the evolution of malaria incidence strongly depends on environmental and socio-demographic characteristics and on the type of interventions used^14–18^. The WHO Framework for Malaria Elimination^19^ recommends that these countries adapt control interventions to suit the local epidemiology and available financial resources. This document also advises using incidence or prevalence as a measure of the malaria burden and suggests dividing the latter into 4 categories of transmission intensity (high, moderate, low, and very low). As a high incidence country, Mali has adopted the WHO’s High Burden to High Impact (HBHI) initiative^10,20^, a targeted malaria response to be led by the health actors of each country. This initiative, which draws on the WHO’s Global Technical Strategy for Malaria 2016–2030 and Sustainable Development Goals on Health, aims to reduce malaria mortality through the implementation of targeted interventions in high burden areas. In Mali, this initiative is also tailored to the priorities of the national malaria strategic plan.

Since 2016–2017, the WHO technical guidelines recommend using malaria risk stratification to better target malaria control interventions. Malaria risk stratification is defined as the classification of geographical areas according to epidemiological, entomological, environmental, and socio-economic factors that determine susceptibility and vulnerability to malaria transmission^10^. Each defined stratum is composed of health districts with similar malaria incidence patterns. The stratification of malaria risk in Mali was first performed in 1989. Five epidemiological strata were identified: (1) the Sudanese-Guinean zone, with long seasonal transmission (4–6 months); (2) the Sahelian zone, with short seasonal transmission (3–4 months); (3) the Saharan zone in the north and some localities of Koulikoro, Segou, Mopti, and Kayes regions, with sporadic or epidemic transmission; (4) dams and the inner delta of the Niger River, with bi or multimodal transmission; and (5) urban zones (especially Bamako and Mopti), with hypoendemic malaria transmission^21,22^. However, the distribution of malaria transmission has considerably evolved over the last 30 years due to climate change, population movement, urbanization, and the impact of malaria control interventions. Accordingly, a new stratification of malaria risk is needed.

This study aimed to perform malaria risk stratification at the health district level in Mali with a view to proposing targeted control interventions.

## Method

### Study location

**Figure 1:**
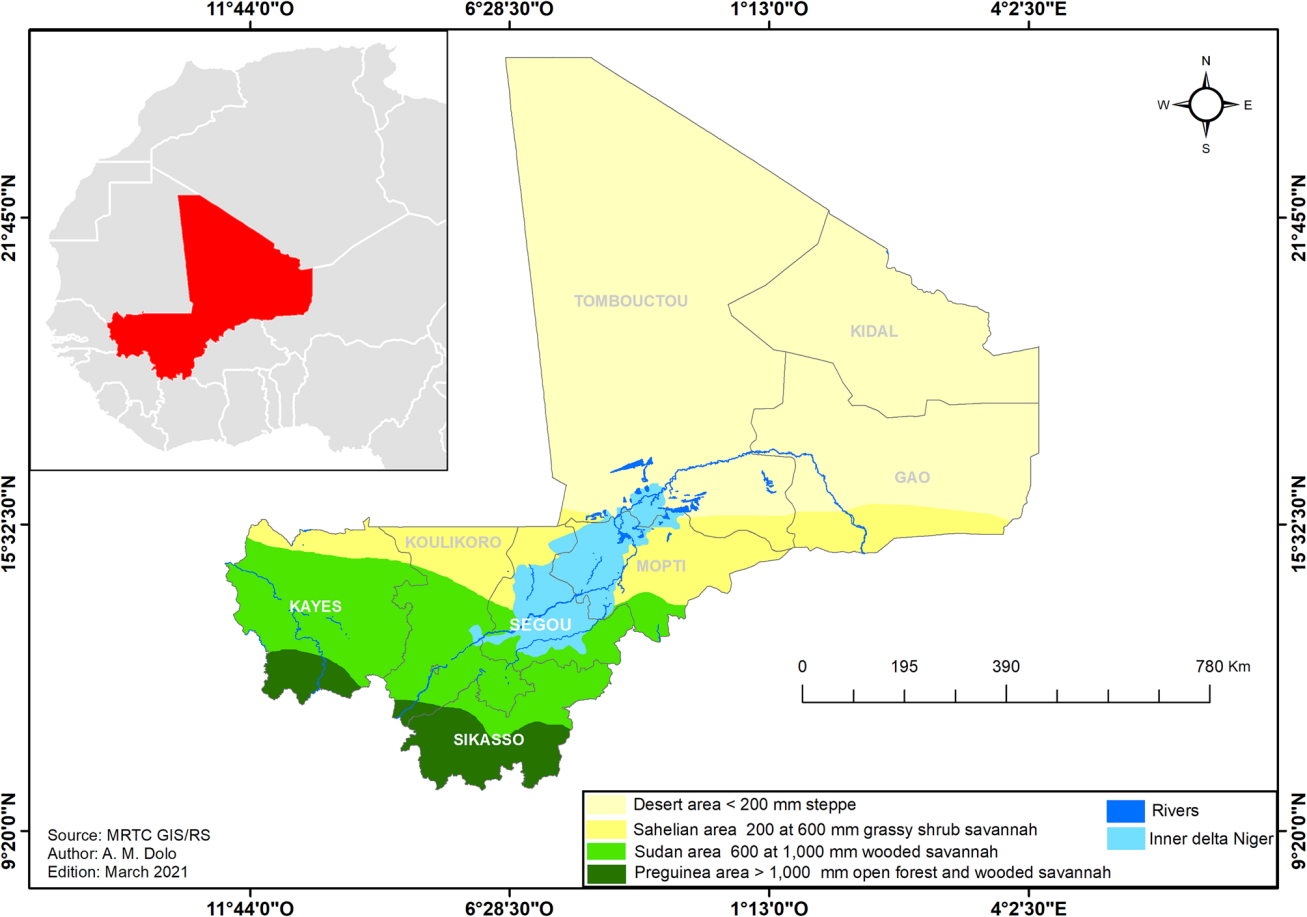
Ecoclimatic zones of Mali (Source: MRTC GIS/RS).

The Republic of Mali is located in the Sahel-Saharan strip in West Africa. In 2020, the country had 20,536,999 inhabitants and a population growth rate of 3.36% (General Population Housing Census (GPHC) 2009 data updated by the National Statistical Institute). The landscape is characterized by plains and low plateaus, with an average altitude of 500 m. The hydrographic regime is constituted by the upper Senegal and Niger Rivers basins. Mali has several dams and flooded areas used for agriculture. Flooded areas extend over an area of 41,000 km^2^ along the Niger River. These areas include the inner delta of the Niger River (from Segou region in the center through Mopti region to Timbuktu region in the north) as well as numerous lakes, ponds, and swamps^23,24^ (Fig. 1).

Mali is divided into 11 regions (including the District of Bamako) and 75 health districts. The health care system is organized in a 3-level pyramid (health district level, regional level, and national level).

### Malaria, child mortality, entomological and environmental data

Data on confirmed malaria cases at the health district level were obtained from the District Health Information Software 2 (DHIS2). National data on malaria prevalence in children aged 6–59 months and mortality in children aged 6–59 months were obtained from the 2018 Demographic and Health Survey (DHS).

Entomological data were obtained from Malian research institutions^6,7,9,25^ that study malaria in the sentinel sites of the NMCP.

Remote sensing environmental data were obtained from the National Aeronautics and Space Administration. These data were: rainfall, relative humidity, temperature, Normalized Difference Vegetation Index (NDVI), soil moisture and wind speed.

The assembled variables are summarized in Table 1.

**Table 1:** Data assembly.

Variables	Sources	Resolution/temporality	Level	Periods
Population	DHIS2	Annual	Health district	2017–2019
Malaria cases	DHIS2	Monthly	Health district	2017–2019
Health facility access*	DHIS2	Monthly	Health district	2017–2019
Health facility usage rate**	DHIS2	Annual	Health district	2017–2019
Rainfall	IMERG v6	Monthly	Health district	2017–2019
Relative humidity	AIRS	Monthly	Health district	2017–2019
Temperature	MERRA-2	Monthly	Health district	2017–2019
Wind speed	GLDAS	Monthly	Health district	2017–2019
Soil moisture	MERRA-2	Monthly	Health district	2017–2019
NDVI	MODIS Terra	Monthly	Health district	2017–2019
Malaria prevalence	DHS	5-Years	Region	2018
Child Mortality***	DHS	5-Years	Region	2018
Entomological data	Research institutions (Sentinel sites)	Annual	15 health districts^⊤^	2010, 2015, 2016, 2017, and 2019

*NDVI* normalized difference vegetation index, *DHIS2* district health information software 2, *IMERG* integrated multisatellite retrievals for GPM, *AIRS* atmospheric infrared sounder, *MERRA-2* modern-era retrospective analysis for research and applications, version 2, *MODIS* moderate resolution imaging spectroradiometer, *GLDAS* global land data assimilation system, *DHS* demographic and health survey, Research institutions: *MRTC* Malaria Research and Training Center, *LBMA* Laboratory of applied molecular biology, *INSP* National Institute of Public Health.

*Health facility access is the proportion of the population living within a radius of 5 km from a health facility, which corresponds to a 60-min walking distance.

**Health facility usage rate is the ratio of new consultations to the total population.

***Mortality in children aged 6–59 months for the period 2012–2017 as per the 2018 DHS.

^⊤^Sentinel sites: Koulikoro (Tienfala), Kati (Dandoly), Kankass (Socoura), Djenné (Madiama), Mopti (Tongorongo), Tominian (Ouena), Bamako, Bla, Sikasso, Bougouni, Selingue, Niono, Kayes, and Kita.

### Data assembly

See Table 1.

### Data analysis

Temporal and spatial analyses were performed using a wide range of variables to account for the complexity of malaria transmission in Mali.

#### Temporal analysis

##### Seasonality of malaria transmission

To determine the periods of low and high malaria transmission, a change point analysis of monthly incidence data was performed using the algorithm of the Power of the Pruned Exact Linear Time^26,27^.

Monthly malaria incidence was estimated using confirmed malaria cases obtained from the DHIS2, taking into consideration population size and time (month).

1 $$malaria \;incidence = \frac{cases}{{population \times time}} \times 1000{ }$$

##### Principal component analysis of environmental variables

A principal component analysis (PCA) of the environmental variables associated with malaria transmission was performed to provide a contextual description of the epidemiological facies of malaria in Mali. These variables were: rainfall, relative humidity, soil moisture, NDVI, and wind speed. The analysis accounted for collinearities between variables and led to the creation of synthetic environmental indices (SEIs)^29^. The number of SEIs was selected using the elbow criterion^28^.

##### Univariate and multivariate regression analyses of the relationship between synthetic environmental indices and malaria transmission

Univariate and multivariate regression analyses of the relationship between SEIs and malaria incidence were performed using Generalized Additive Models (GAMs), taking into consideration the lag between the two^30^. Spline smoothing was performed to study the non-linear relationship between malaria incidence and SEIs. A negative binomial distribution was conducted to adjust for over-dispersion. The time series of monthly malaria incidence was modeled as a function of SEIs with a lag of 1–4 months.

#### Spatial analysis

##### Spatial heterogeneity of malaria incidence

Crude estimations of malaria incidence (see the Seasonality of malaria transmission sub-section above) do not fully reflect the real incidence of the disease in the population, as the following factors vary between health districts^29^:

- Health facility access and usage rate.
- Availability and use of diagnostic tests.
- Completeness of the data.

In view of this, incidence was estimated per 1000 person-years adjusting for the health facility usage rate:

2 $$adjusted\; malaria \;cases = \left( {\frac{confirmed \;cases}{{health\; facility \;usage\; rate}}} \right) \times 100$$

3 $$adjusted \;malaria\; incidence = \frac{adjusted \;cases}{{population \times time}} \times 1000{ }$$

Given that the use of diagnostic tests depends on health facility access and usage, adjustment for health facility usage indirectly accounted for the testing rate. There was no need to adjust for data completeness because the completeness rate remained stable (at around 98%) over the study period^30^.

Based on adjusted malaria incidence, the 75 health districts of Mali were classified into 4 categories of transmission intensity (high, moderate, low, and very low) in accordance with the WHO Framework for Malaria Elimination^19^. The very low category was divided into two sub-categories to better reflect incidence in health districts where the risk of malaria transmission is close to zero.

##### Spatial distribution of rainfall

The spatial distribution of mean annual rainfall in the health districts of Mali over the period 2017–2019 was analyzed.

##### Spatial distribution of health facility access

The spatial distribution of health facility access in all health districts of Mali in the year 2019 was analyzed.

##### Spatial distribution of prevalence in children aged 6–59 months

The spatial distribution of the prevalence of malaria in children aged 6–59 months in Mali in the year 2018 was analyzed.

##### Spatial distribution of mortality in in children aged 6–59 months

The spatial distribution of mortality in children aged 6–59 months in Mali over the period 2012–2017 was analyzed.

##### Spatial distribution of vector resistance

Two parameters were examined: vector distribution and vector resistance to 10 insecticides from 5 classes: permethrin 0.75%, deltamethrin 0.05%, alpha-cypermethrin 0.05%, and lambda-cyhalothrin 0.05% (pyrethroids); DDT 4% (organochlorines); bendiocarb 0.1% and propoxur 0.1 (carbamates); pirimiphos-methyl 0.25% and 1% and fenitrothion 1% (organophosphates); and clothianidin 2% (neonicotinoids). The resistance of the main vector species, *An. gambiae*, was measured using bioassays as per WHO recommendations.

### Selection of interventions

In accordance with the WHO Global Technical Strategy for Malaria 2016–2030^9^, the WHO HBHI initiative^20^, the WHO Framework for Malaria Elimination^19^, the national health priorities for malaria control, and the national malaria strategic plan, a package of interventions was discussed and qualitatively selected with the NMCP and the different funding partners. Depending on the type of intervention, health districts were targeted based on the most relevant variables, the impact of each intervention on the burden of malaria (HBHI), the cost of the intervention, and the resources available for the intervention.

For the purpose of intervention selection, variables were categorized as follows:

- Malaria incidence and malaria prevalence were classified into 4 categories of transmission intensity (high, moderate, low, and very low) in accordance with the WHO Framework for Malaria Elimination.
- Mortality in children aged 6–59 months was dichotomized into high or low. The threshold value was 101 per 1000 live births, which corresponds to the national average estimated in 2019.
- Health districts were classified as vector resistant or not, depending on whether they showed confirmed *An. gambiae* resistance to pyrethroids.
- Health facility usage rate was dichotomized into high or low. The threshold value was 58%, which corresponds to the national average estimated in 2019.

The resources available for interventions consisted in grants from funding partners and funds allocated in the NMCP national budget for malaria control over the period 2021–2023.

The targeting of interventions is detailed in the Selected interventions section below.

### Software and packages

Statistical analyses were carried out with R software version 3.4 (R Development Core Team, R Foundation for Statistical Computing, Vienna, Austria). The following packages were used: {mgcv}{caschrono}{FactoMineR}{forecast}{ggplot2}{party}{changepoint}.

Mapping was performed using ArcGIS Desktop version 10.1 software (Environmental Systems Research Institute (ESRI), 380 New York Street, Redlands, CA 92373-8100, USA).

The Paint.net software version 4.2.13 (Rick Brewster and al., Washington State University, USA) was used for image treatment.

### Consent to publication

Not applicable.

## Results

### Temporal analysis

#### Seasonality of malaria transmission

**Figure 2:**
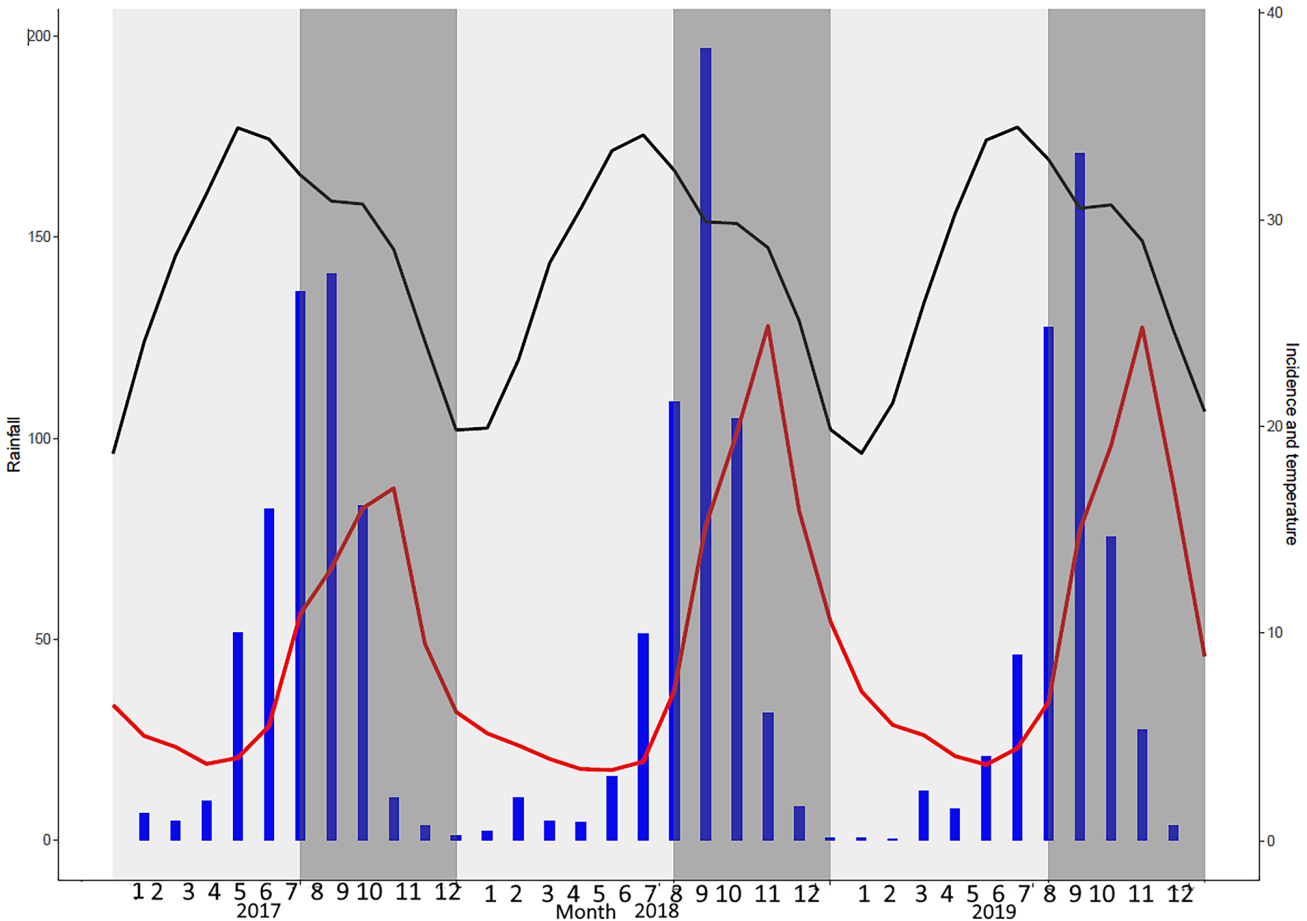
Malaria incidence time series, Mali, 2017–2019. Grey band: periods of high malaria transmission; Blue bars: rainfall (left y-axis, mm); Red curve: malaria incidence (y-right axis, cases/person-months); Black curve: average monthly temperature (y-right axis, °C).

The descriptive analysis of monthly malaria incidence time series showed typical seasonality at the national level, with a lag between cases and rainfall (Fig. 2).

#### Principal component analysis of environmental variables

**Figure 3:**
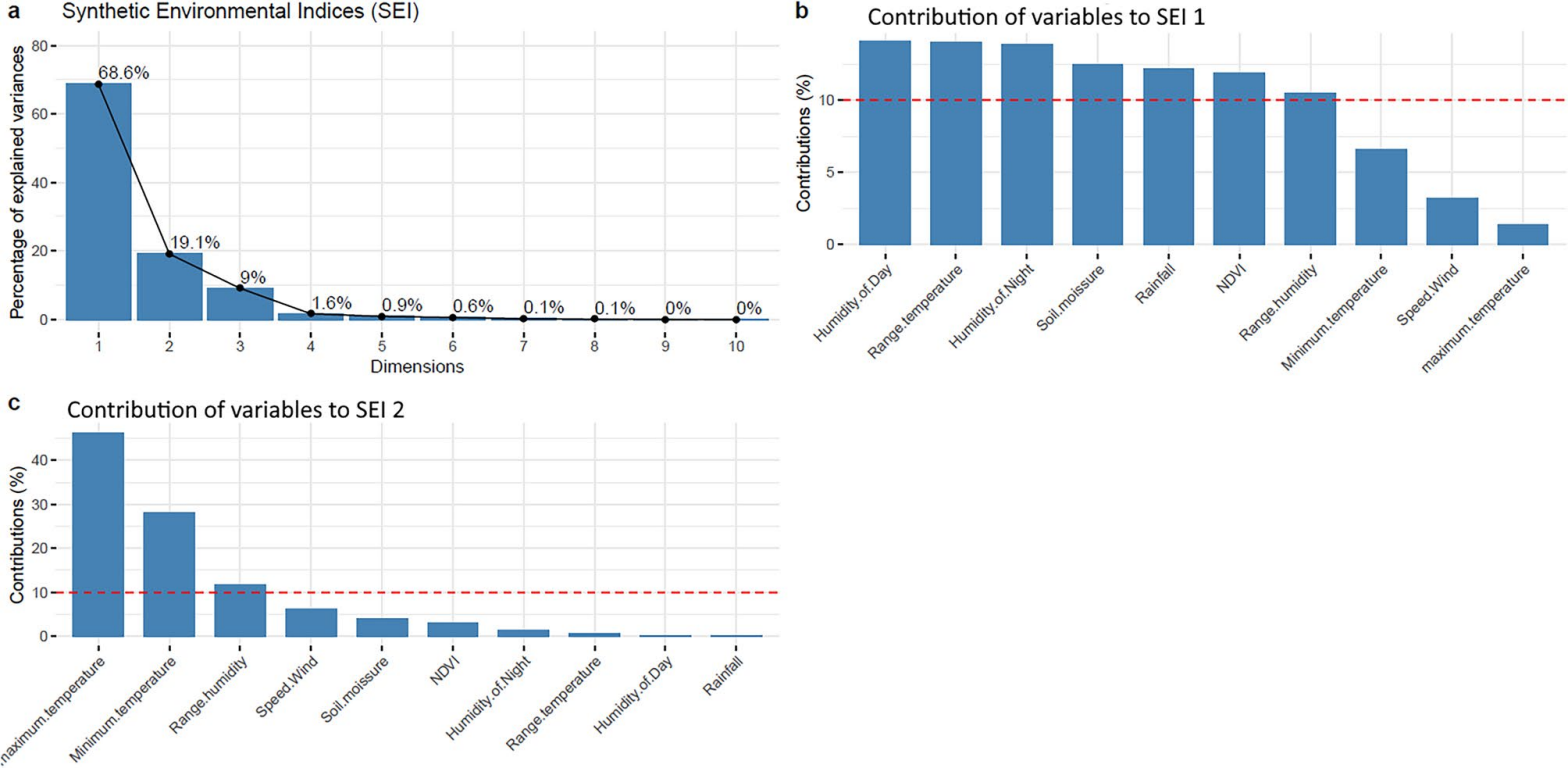
Synthetic indices resulting from the Principal Component Analysis of the different environmental variables. (**Panel a**) Two Synthetic Environmental Indices with 87.7% inertia. (**Panel b**) Synthetic Environmental Index 1 (rainfall, relative humidity, soil moisture, Normalized Difference Vegetation Index, and wind speed). (**Panel c**) Synthetic Environmental Index 2 (temperature).

The PCA analysis yielded 2 SEIs (Fig. 3).

#### Univariate and multivariate regression analyses of the relationship between synthetic environmental indices and malaria transmission

Univariate regression analysis using GAM found a 1-month lag between SEI 1 (rainfall, relative humidity, soil moisture, NDVI, and wind speed) and malaria incidence. No lag was found between SEI 2 (temperature) and malaria incidence.

**Figure 4:**
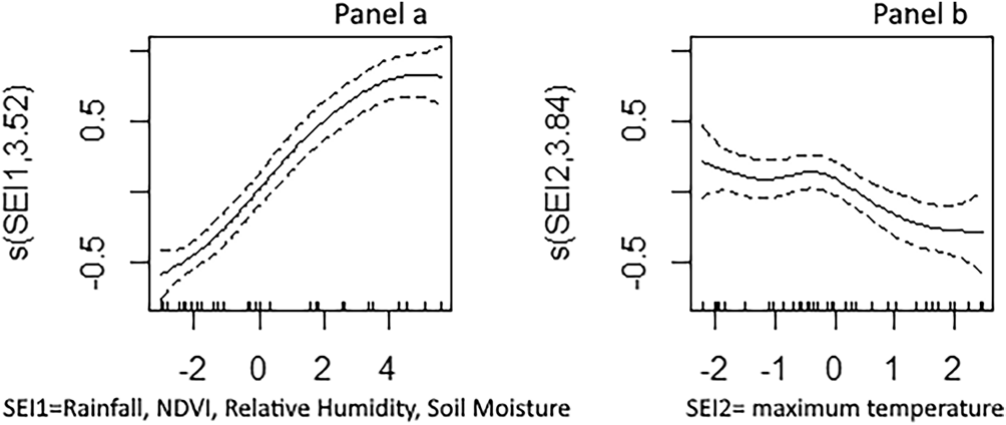
Changes in malaria incidence as a function of synthetic environmental indices; multivariate regression analysis using a Generalized Additive Model. (**Panel a**) Positive linear relationship between SEI 1 (rainfall, relative humidity, soil moisture, NDVI, and wind speed) and malaria incidence (*p* value < 0.001). (**Panel b**) Negative non-linear relationship between SEI 2 (temperature) and malaria incidence (*p* value < 0.01).

Multivariate regression analysis using GAM (Fig. 4) showed a positive linear relationship between SEI 1 (rainfall, relative humidity, soil moisture, NDVI, and wind speed) and malaria incidence (*p* value < 0.001) (Fig. 4, panel a) and a negative non-linear relationship between SEI 2 (temperature) and malaria incidence (*p* value < 0.01) (Fig. 4, panel b).

### Spatial analysis

#### Spatial heterogeneity of malaria incidence

Adjusted median incidence for the year 2019 was 125.34 cases per 1000 person-years with a standard deviation of 86.48.

**Figure 5:**
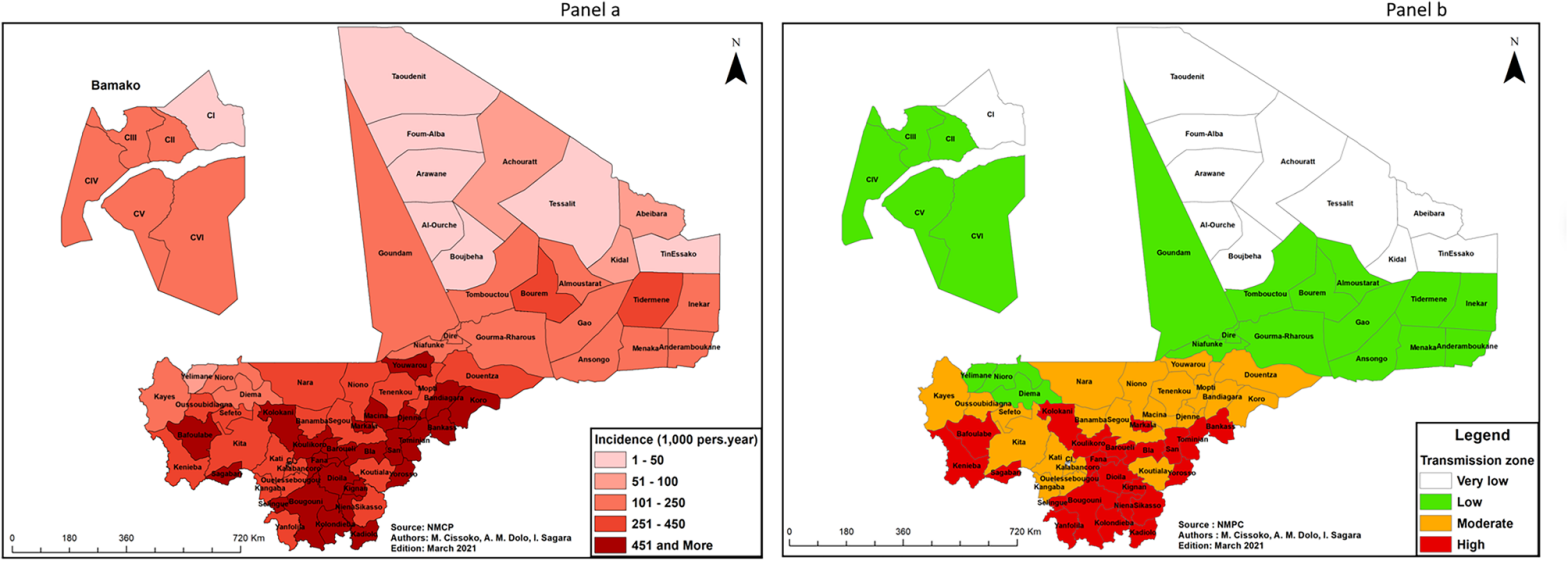
Annual malaria incidence adjusted for health facility usage rate in 75 health districts Mali, 2019. ArcGIS Desktop version 10.8 software (Environmental Systems Research Institute (ESRI), 380 New York Street, Redlands, CA 92373-8100, USA, https://www.esri.com).

In accordance with WHO recommendations, adjusted malaria incidence per 1000 person-years for the year 2019 was classified into 4 categories of transmission intensity (Fig. 5):

- 12 health districts were located in areas of very low malaria transmission (less than 100 cases per 1000 person-years), including 7 health districts with 5–50 cases per 1000 person-years (in the northern regions) and 5 health districts with 50–100 cases per 1000 person-years (3 in the northern regions, 1 in the western part of the country, 1 in the District of Bamako);
- 19 health districts were located in areas of low malaria transmission (between 100 and 250 cases per 1000 person-years);
- 20 health districts were located in areas of moderate malaria transmission (between 250 and 450 cases per 1000 person-years).
- 24 health districts were located in areas of high malaria transmission (450 or more cases per 1000 person-years) (mainly in the southern and central-western regions).

#### Spatial distribution of rainfall

**Figure 6:**
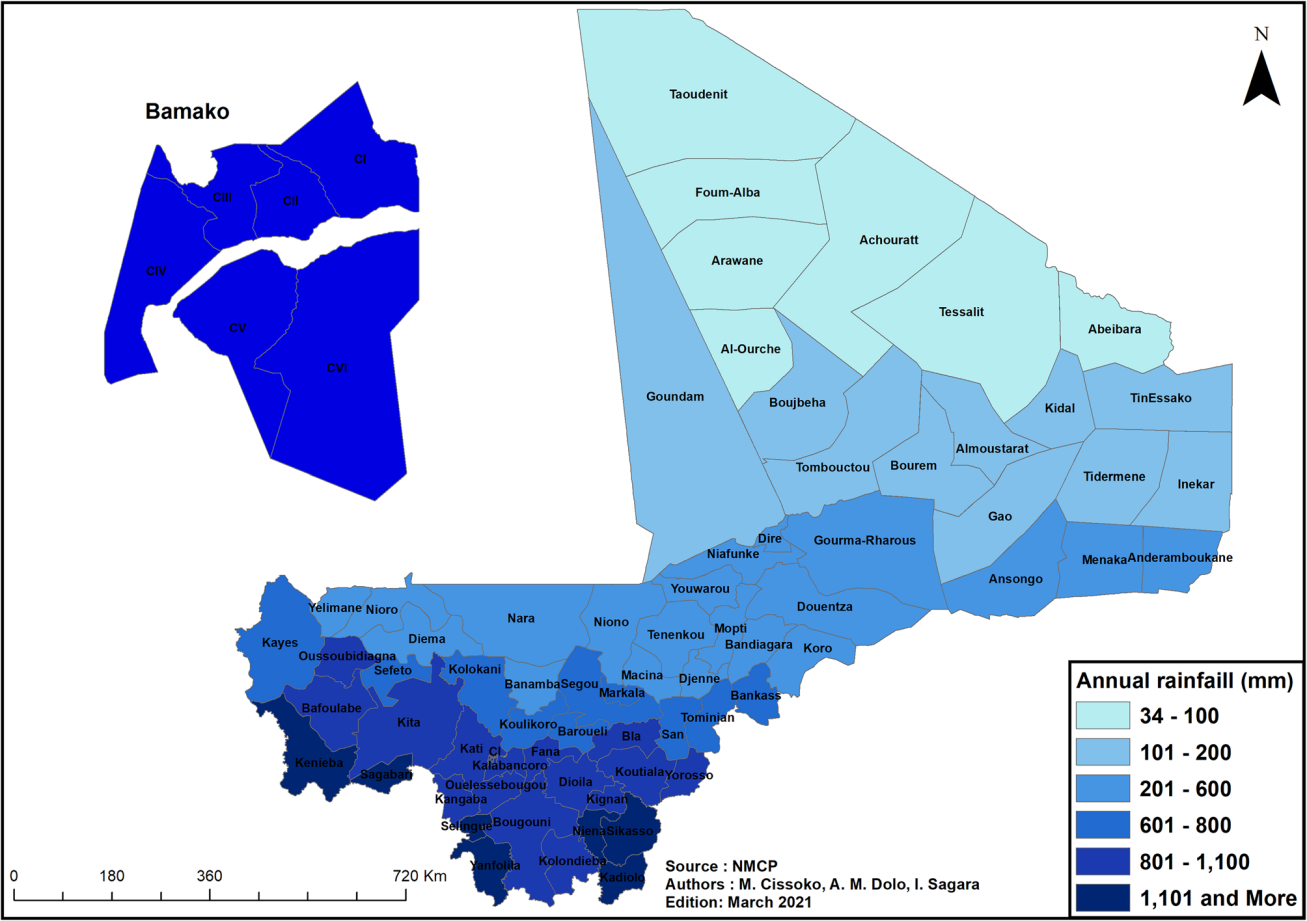
Map of average annual rainfall by health district, Mali, 2017–2019. ArcGIS Desktop version 10.8 software (Environmental Systems Research Institute (ESRI), 380 New York Street, Redlands, CA 92373-8100, USA, https://www.esri.com).

Average annual rainfall over the 2017–2019 period ranged from less than 200 mm in the Saharan zone to more than 1100 mm in the pre-Guinean zone, with a clear trend from north to south. Of the 75 health districts, 17 had an average annual rainfall of less than 200 mm (Saharan zone), 20 had an average annual rainfall between 200 and 600 mm (Sahelian zone), 31 had an average annual rainfall between 600 and 1100 mm (Sudanese zone), and 7 had an average annual rainfall > 1100 mm (pre-Guinean zone) (Fig. 6).

#### Spatial distribution of health facility access

**Figure 7:**
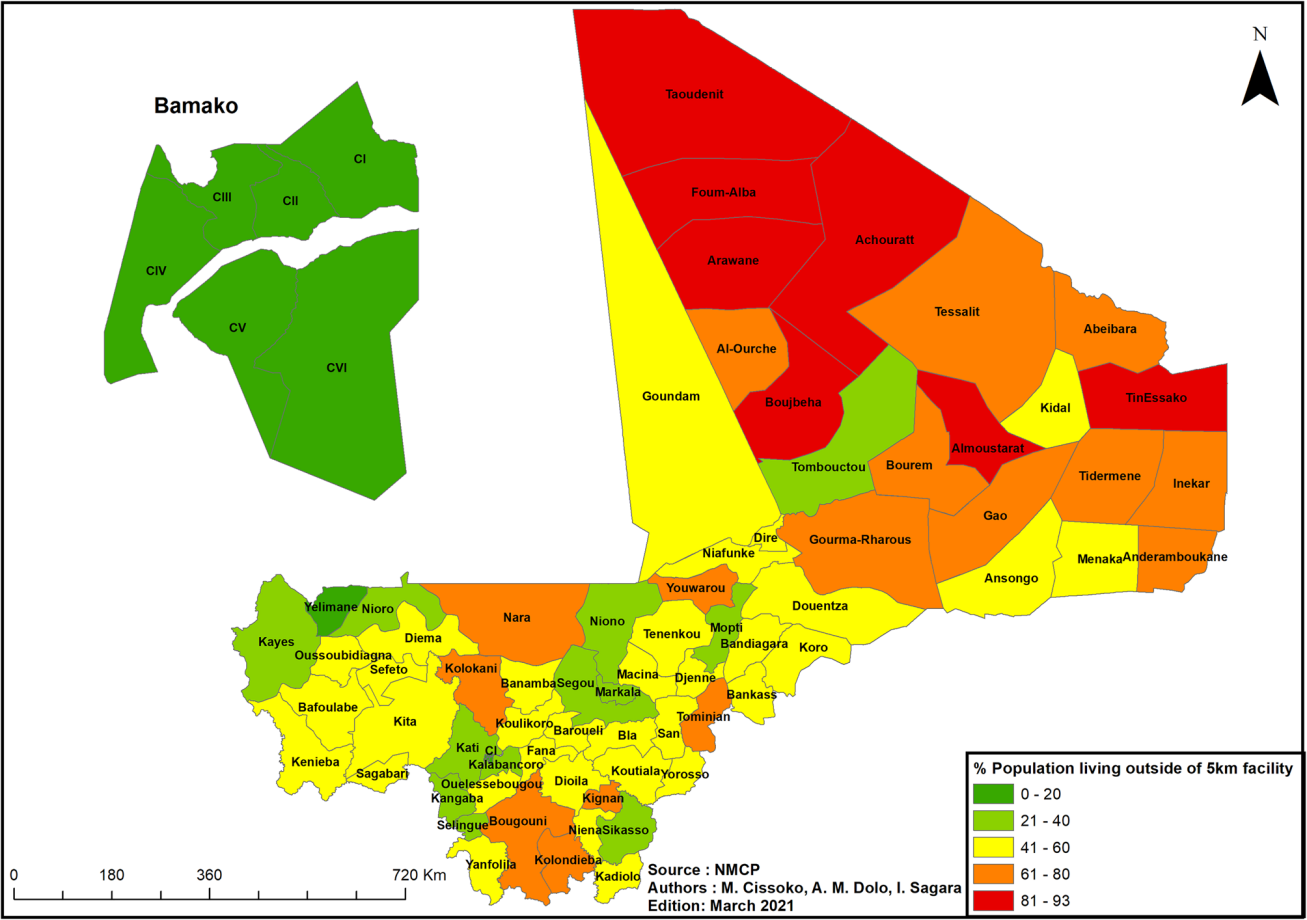
Percentage of the population living outside a radius of 5 km from a health facility access, Mali, 2019. ArcGIS Desktop version 10.8 software (Environmental Systems Research Institute (ESRI), 380 New York Street, Redlands, CA 92373-8100, USA, https://www.esri.com).

The percentage of the population living outside a radius of 5 km (Euclidian distance) from a health facility was high (especially in the northern regions), justifying the implementation of specific strategies to improve health facility access (Fig. 7).

Health facility access was low in health districts with high malaria transmission. One strategy for improving access to malaria diagnosis is the conduct of RDTs at the community level via integrated community case management (iCCM).

#### Spatial distribution of prevalence of malaria in children aged 6–59 months

**Figure 8:**
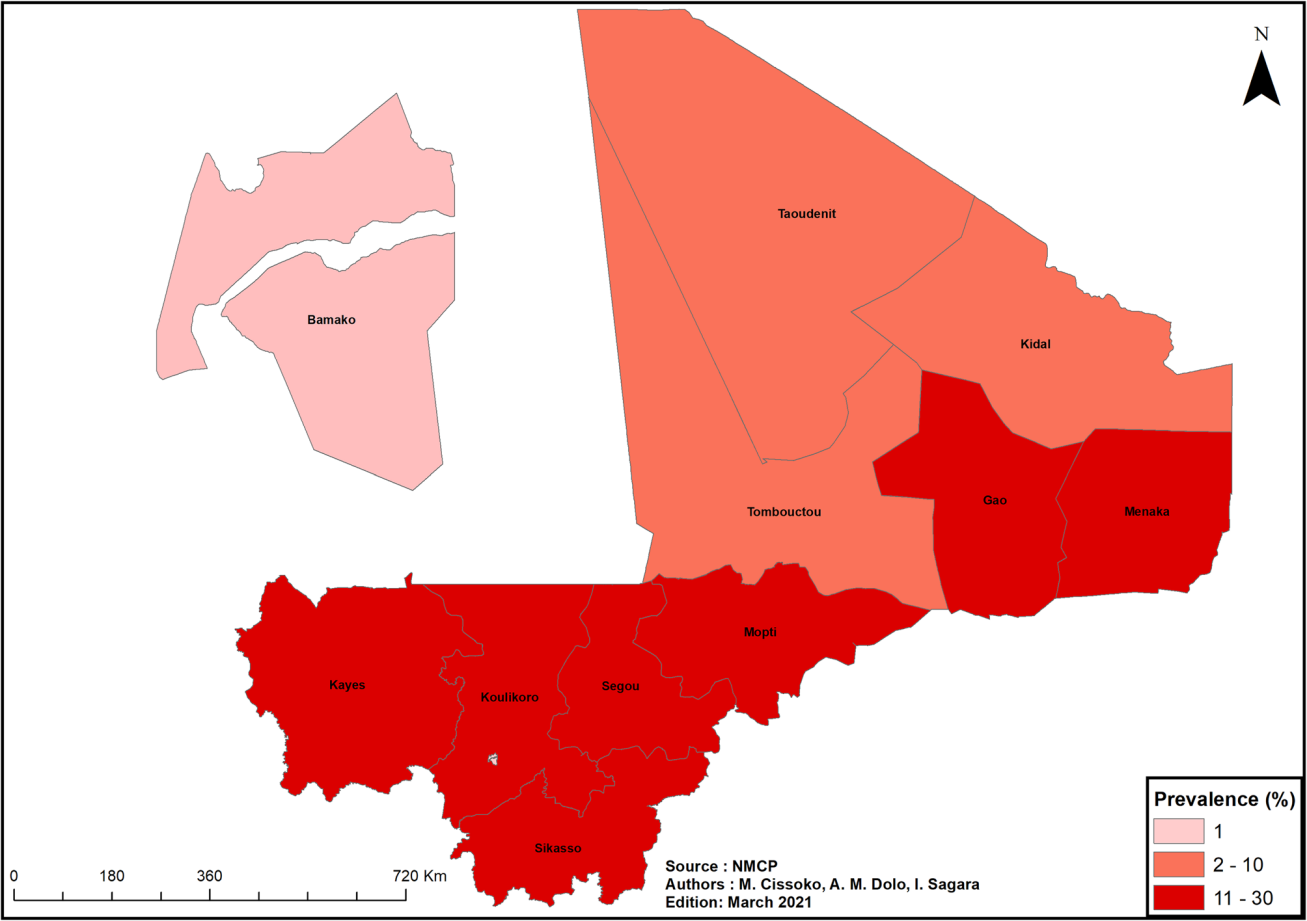
Map of the prevalence of malaria in children aged 6–59 months, Mali, 2018. ArcGIS Desktop version 10.8 software (Environmental Systems Research Institute (ESRI), 380 New York Street, Redlands, CA 92373-8100, USA, https://www.esri.com).

According to the Mali DHS VI, the prevalence of malaria in children aged 6–59 months was 18.9% at the national level in 2018. This prevalence was lower in the northern regions of Kidal, Taoudenit, and Tombouctou (between 1 and 10%) and in the District of Bamako (less than 1%). The regions of Sikasso, Segou, and Mopti had the highest prevalence (29.7%, 25.9%, and 24.9%, respectively) (Fig. 8).

#### Spatial distribution of mortality in children aged 6–59 months

**Figure 9:**
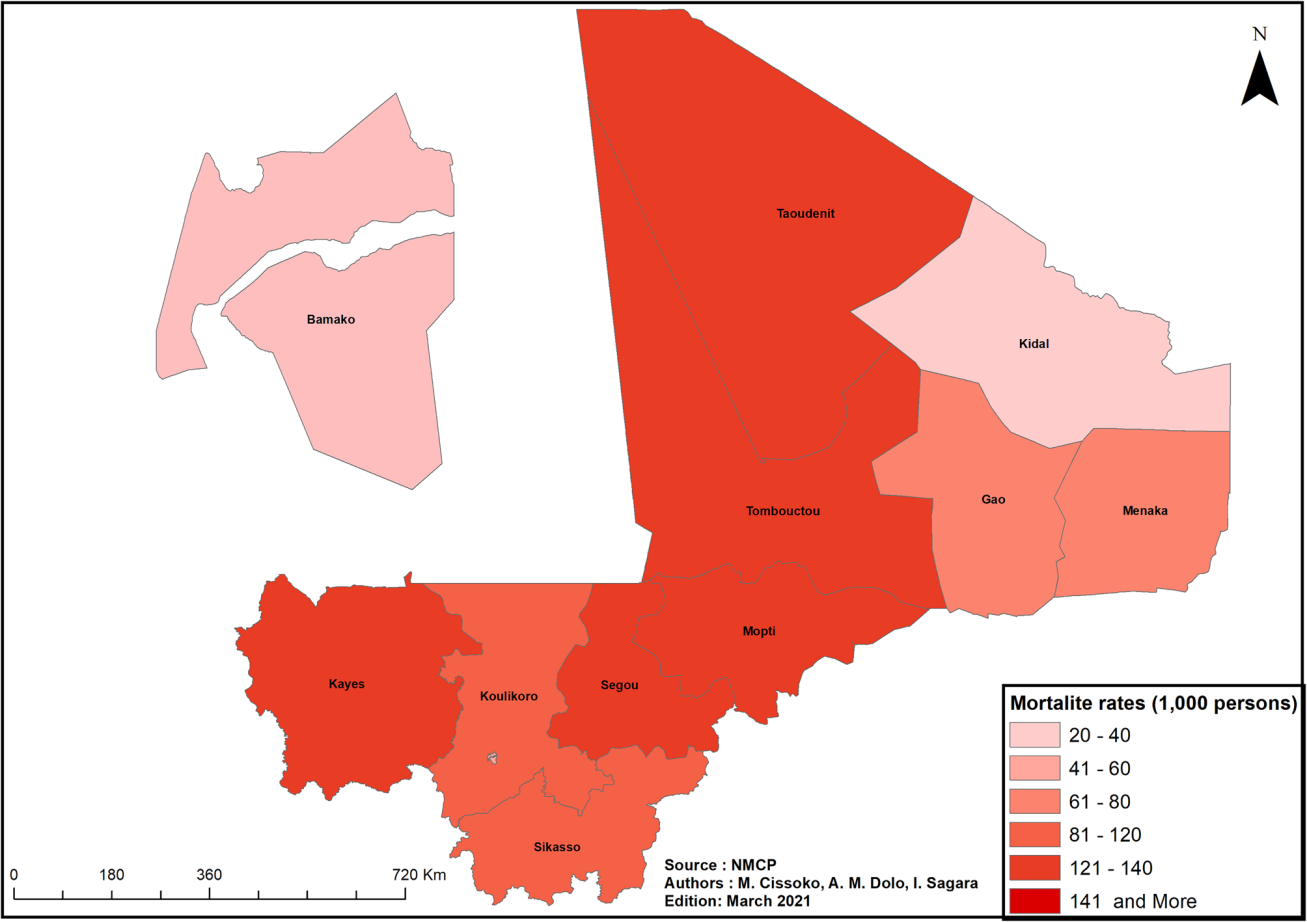
Map of mortality for children aged 6–59 months, Mali, 2018.

Kidal region, Gao region, and the District of Bamako had the lowest child mortality (20, 78, and 55 deaths per 1000 live births, respectively) (Fig. 9).

#### Spatial distribution of vector resistance

**Figure 10:**
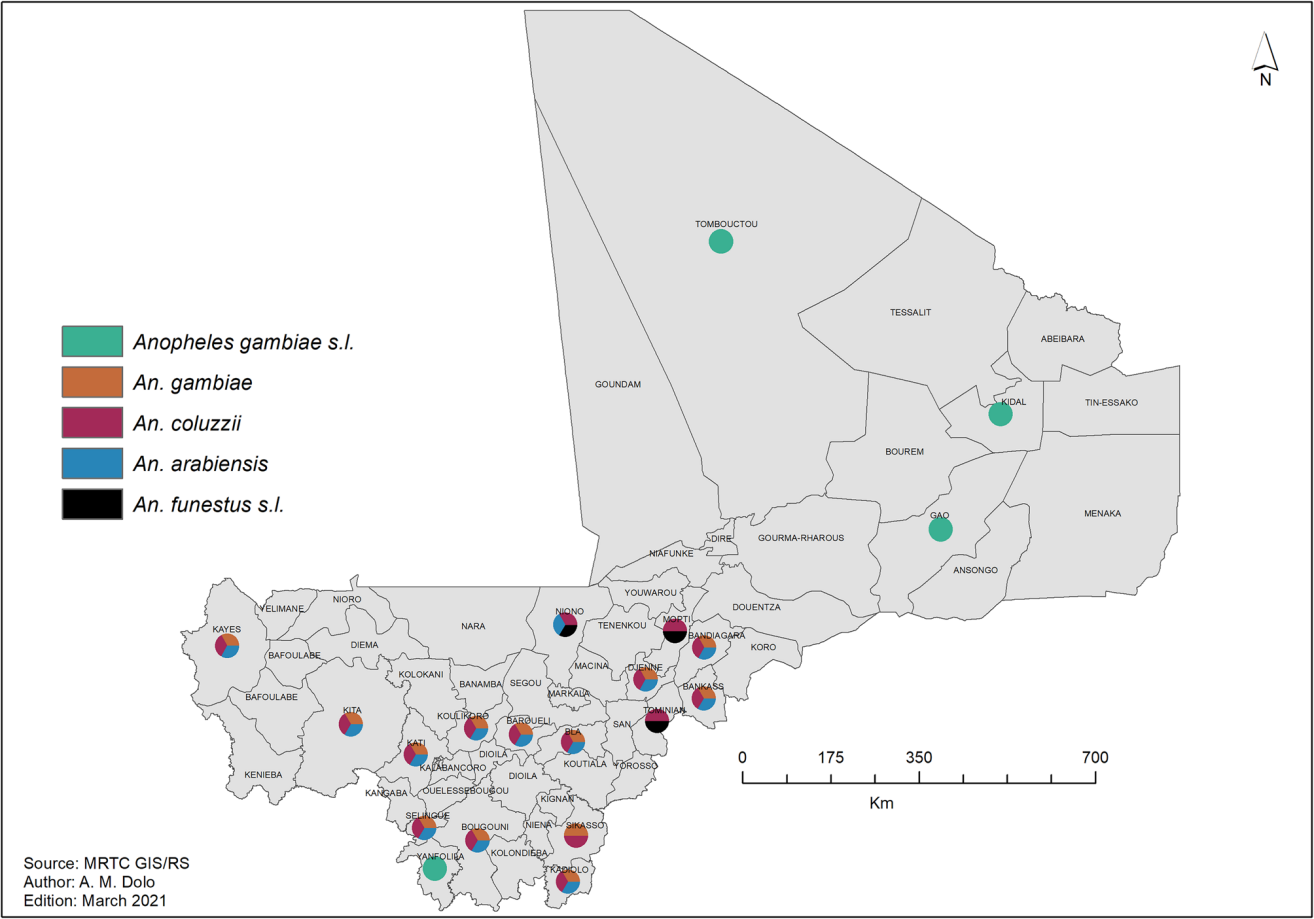
Map of malaria vector distribution, Mali, 2010–2019. The sector diagrams show the presence of different vector species. ArcGIS Desktop version 10.8 software (Environmental Systems Research Institute (ESRI), 380 New York Street, Redlands, CA 92373-8100, USA, https://www.esri.com).

The main malaria vector species—*An. gambiae*, *An. coluzzii,* and to a lesser extent *An. Arabiensis*—were found in all sentinel sites using morphological analysis. Only *An. gambiae* was detected in the northern regions of Gao, Kidal, and Tombouctou. *An. funestus* was identified mainly in the regions of Niono and Mopti (inner delta of the Niger River) (Fig. 10).

**Figure 11:**
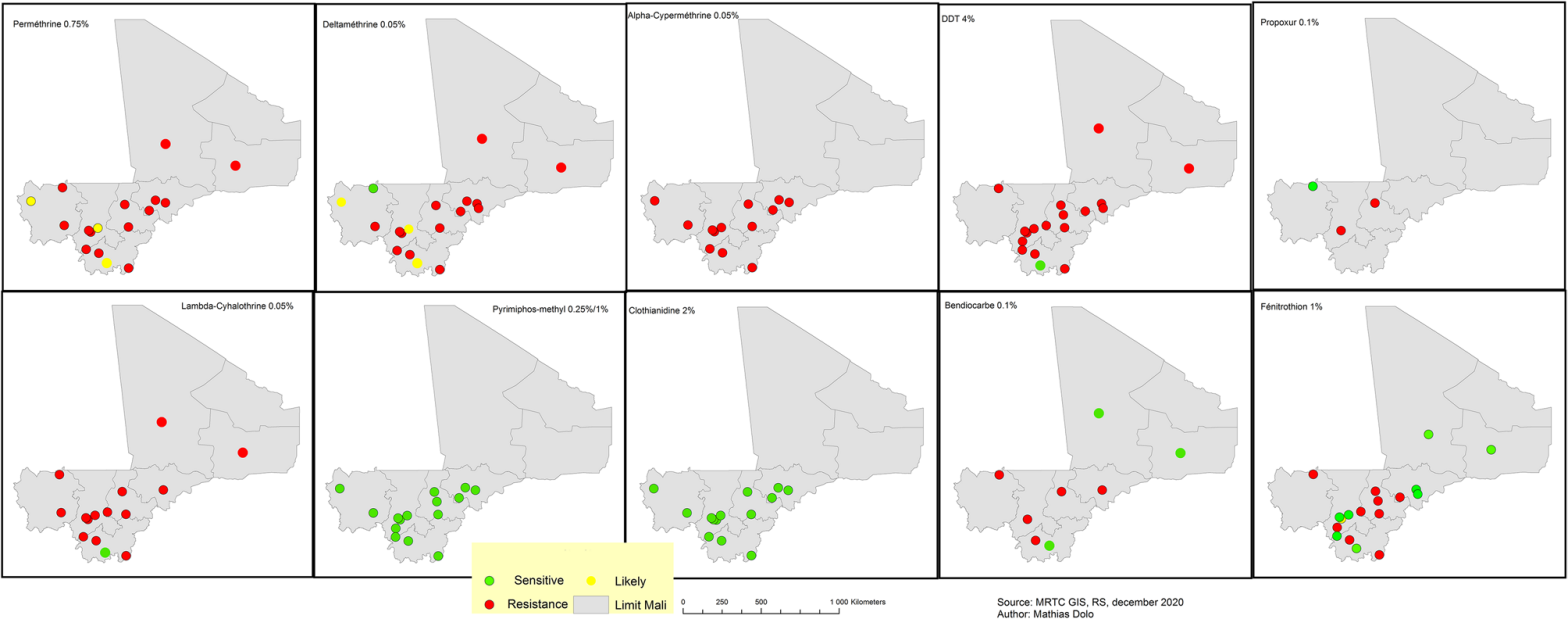
Map of distribution of vector resistance in sentinel sites, Mali, 2010–2019. Red dots represent vector resistance; yellow dots show moderate sensitivity (below 90%); and green dots show high sensitivity (above 90%). ArcGIS Desktop version 10.8 software (Environmental Systems Research Institute (ESRI), 380 New York Street, Redlands, CA 92373-8100, USA, https://www.esri.com).

The main vector species, *An. gambiae*, showed high resistance to pyrethroids and organochlorines in most sentinel sites. Resistance to carbamates (bendiocarb and propoxur) was also noted, although it was less extensive than resistance to pyrethroids. *Anopheles gambiae* was sensitive to pirimiphos-methyl and clothianidin in all sentinel sites. Depending on the site, it showed sensitivity or resistance to fenitrothion (Fig. 11).

### Selected interventions

Potential control interventions were discussed with the NMCP and the different funding partners. Eight intervention combinations were qualitatively selected for implementation in the different health districts based on the following variables: spatial heterogeneity of malaria incidence, prevalence of malaria in children aged 6–59 months, distribution of vector resistance, health facility usage, mortality in children aged 6–59 months, and seasonality of transmission. The impact of each intervention on the burden of malaria (HBHI), the cost of the intervention, and the resources available for the intervention were also taken into consideration. Environmental variables were not considered in discussions with the NMCP but were indirectly accounted for as they are correlated with malaria incidence in Mali (see Figs. 4, 5 and 6).

The 8 intervention combinations are made up of the following interventions:

*Long-lasting insecticidal net distribution* will be implemented on a large scale based on the relatively low *cost of the intervention* and the *endemicity of malaria* in Mali. This intervention will be carried out using 2 methods. In the first method, LLINs will be routinely distributed in all 75 health districts to 9-month-old children vaccinated against measles via the Expanded Programme on Immunization and to pregnant women during prenatal consultations. In the second method, LLINs will be distributed in 69 health districts as part of the 2023 universal coverage campaign. Six health districts located in the urban area of Bamako will be excluded because of low malaria prevalence in children aged 6–59 months^2^. The distribution of LLINs in these 69 health districts will be renewed every 3 years as part of the universal coverage campaign^31^.

Different types of LLINs will be distributed based on *malaria incidence* and *vector resistance to pyrethroids*. Interceptor G2 (IG2) nets will be distributed in 4 health districts with high malaria incidence and confirmed vector resistance to pyrethroids (Yorosso, Yanfolila, Selingue, and Kadiolo) both routinely and as part of the 2023 universal coverage campaign. Note that these 4 health districts already received IG2 nets in 2020 (pilot phase). Long-lasting piperonyl butoxide-treated insecticidal nets (PBO-LLINs) will be distributed in 14 health districts with high malaria incidence (Bandiagara, Bankass, Bareouli, Bla, Bougouni, Djenne, Fana, Kati, Kayes, Kita, Koulikoro, Mopti, Niono, and Tominian) both routinely and as part of the 2023 universal coverage campaign. In 13 of these health districts, the main vector species, *An. gambiae*, was found to be resistant to pyrethroids. The last health district, Tominian, will receive PBO-LLINs despite the lack of data on vector resistance because it has a high level of malaria incidence and because it is surrounded by health districts that will receive IG2 nets and PBO-LLINs. In the other 57 health districts, for which there is no data on vector resistance, standard LLINs will be distributed routinely; 51 of these health districts (all but the 6 health districts of Bamako) will also receive standard LLINs as part of the universal coverage campaign^2^.

*Seasonal malaria chemoprevention* will be implemented in all but 17 health districts based on *malaria incidence*, *malaria prevalence in children aged 6–59 months (*in the case of Bamako), and *seasonality of transmission*. The 6 health districts of Bamako will be excluded from the intervention due to low malaria prevalence (Fig. S1, Panel b)^2^. The 6 health districts of Taoudenit region along with 3 health districts in Kidal region (Tin Essako, Abeibara, and Tessalit) and 2 health districts in Menaka region (Inekar and Anderamboukane) will be excluded due to very low malaria incidence (less than 100 cases per 1000 person-years) and to the absence of seasonality throughout the year (Fig. S1, Panel a). Based on the duration of the high malaria transmission season, the intervention will be carried out in 4 rounds (over a period of 4 months) in the health districts located in the southern regions (Fig. S1, Panels c, d, e) and in 3 rounds (over a period of 3 months) in the health districts located in the northern regions (Fig. S1, Panel f).

*Intermittent preventive treatment in pregnancy* will be continued in all 75 health districts based on the relatively low *cost of the intervention* and the *endemicity of malaria* in Mali^32^, in accordance with WHO recommendations^33^.

*Indoor residual spraying* will be implemented based on *available resources,* the *cost of insecticides,* and *vector resistance to pyrethroids* in the different health districts. The intervention was initially planned in 11 health districts with high malaria incidence (Bandiangara, Bougouni, Dioila, Douentza, Fana, Kalabancoro, Nara, Niono, Segou, Yanfolila, and Youwarou). However, due to insufficient resources, only the health districts of Bandiagara, Mopti, and Djenne will receive IRS in 2022^7,8,34^. These 3 health districts were selected because they are the most accessible and because they have the highest population density.

*Malaria case management* will be implemented in all 75 health districts based on the relatively low *cost of the intervention* and the *endemicity of malaria* in Mali. This intervention will be performed in accordance with national guidelines and protocols, themselves based on WHO recommendations.

*Integrated community case management* will be implemented in 51 health districts based on *mortality in children aged 6–59 months* and *health facility access* (Fig. 7). Most of the targeted health districts have high or moderate malaria incidence. Existing iCCM sites will be used and new ones created, and all iCCM sites will be provided with additional diagnostic tools. A pilot study of SP distribution to pregnant women will be conducted in 3 health districts.

Lastly, *weekly and monthly epidemiological surveillance* will be continued in all health districts and will be reinforced in the 27 health districts with epidemic potential.

**Figure 12:**
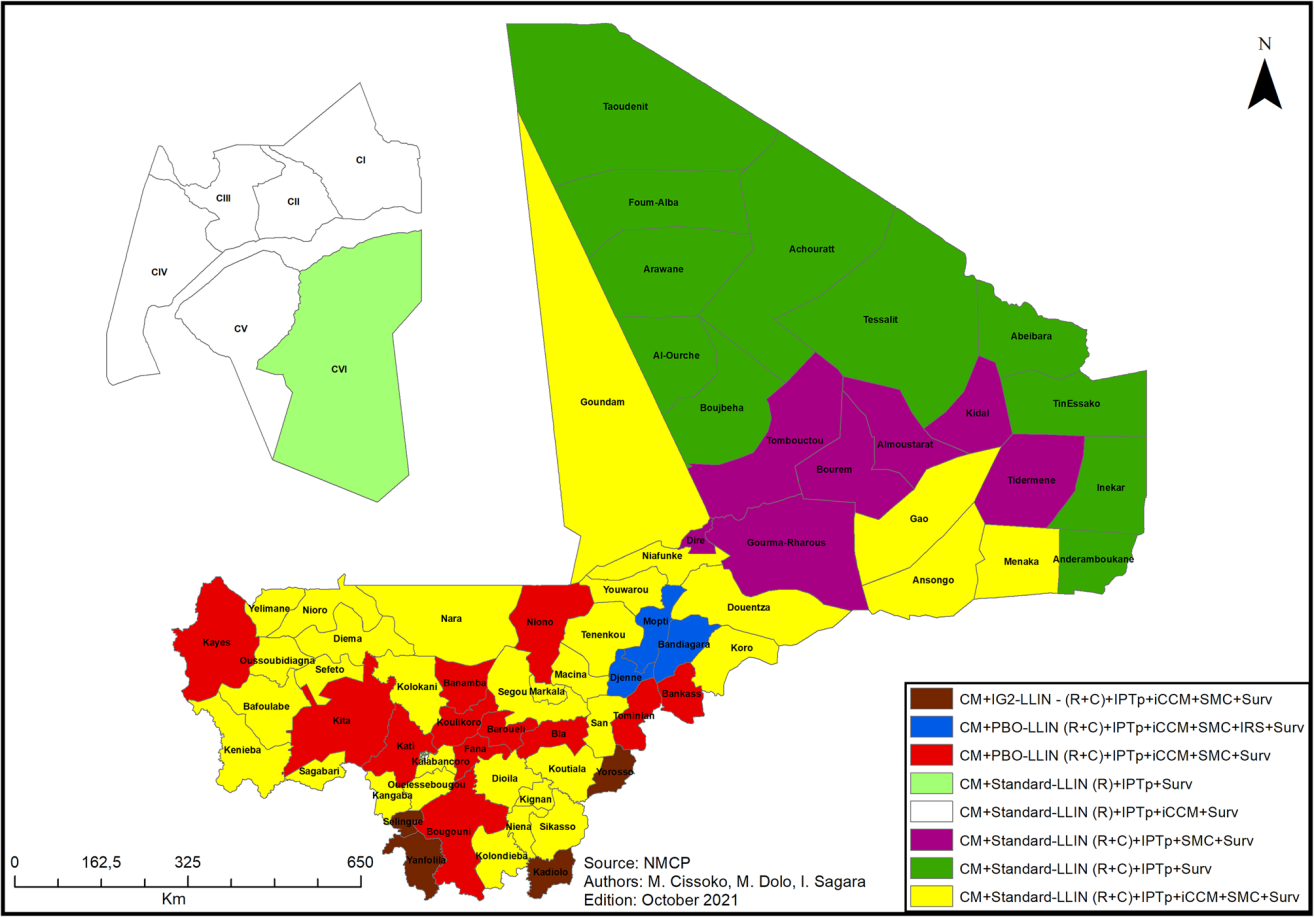
Intervention combinations at the health district level in Mali. CM: Malaria case management; IG2-LLIN: Interceptor G2 Net; R: Routine distribution; C: Universal campaign; IPTp: Intermittent preventive treatment in pregnancy; iCCM: Integrated community case management; SMC: Seasonal malaria chemoprevention; Surv: Weekly and monthly epidemiological surveillance; PBO-LLIN: Long-lasting piperonyl butoxide-treated insecticidal net; IRS: Indoor residual spraying; Standard LLIN: Standard long-lasting insecticidal net. ArcGIS Desktop version 10.8 software (Environmental Systems Research Institute (ESRI), 380 New York Street, Redlands, CA 92373-8100, USA, https://www.esri.com).

Selected intervention combinations at the health district level are shown in Fig. 12. The main interventions (SMC, IG2-LLIN, PBO-LLIN, IRS, IPT, CM) will be implemented in all health districts with high and moderate transmission.

The population targeted by the 8 intervention combinations is shown in Table 2. Note that the colors associated with the different intervention combinations are the same as those used in Fig. 12.

**Table 2:**
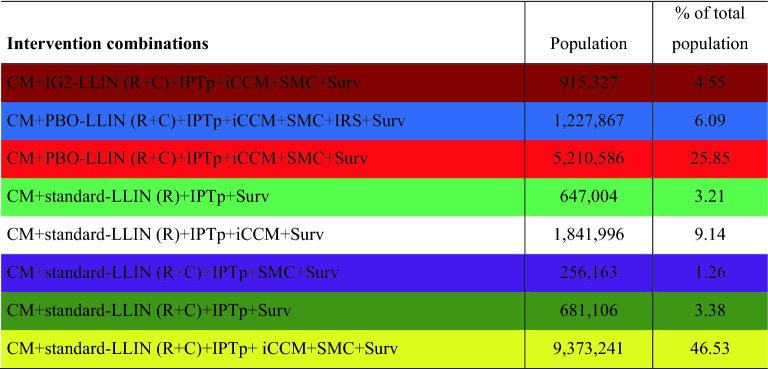
Population targeted by the 8 intervention combinations.

The selected intervention combinations will target more than 78% of the total population of Mali.

## Discussion

In accordance with the WHO Global Technical Strategy for Malaria 2016–2030, the WHO HBHI initiative, the WHO Framework for Malaria Elimination, the national health priorities for malaria control, and the national malaria strategic plan, a package of interventions was discussed and selected with the NMCP and the different funding partners. Interventions were qualitatively selected based on the following variables: spatial heterogeneity of malaria incidence, prevalence of malaria in children aged 6–59 months, distribution of vector resistance, health facility usage, mortality in children aged 6–59 months, and seasonality of transmission. The impact of the intervention on the burden of malaria, the cost of the intervention, and the resources available for the intervention were also taken into consideration. Eight intervention combinations were selected for implementation in the different health districts. The stratification will be revised every 5 years depending on the evolution of malaria incidence and the availability of new data on parasite and vector resistance.

In the absence of prevalence data at the health district level, incidence data were used for the stratification of malaria risk. Because access to care varies according to the number of health facilities and the availability of community health workers, these incidence data were adjusted for the health facility usage rate to estimate the number of effective cases in the population^29,35^. It should be noted that these incidence data were of high quality thanks to the DHIS2 data collection platform and the availability of malaria diagnostic tools, particularly RDTs, at the health district level. Incidence data were relevant for our stratification as they are available for free and allow for measuring the temporal evolution of malaria transmission.

In our study, SEIs were created to measure the impact of environmental factors on malaria transmission, and to thus provide a contextual description of the epidemiological facies of malaria in Mali. These SEIs were not considered in the targeting of interventions but were indirectly accounted for because the spatial heterogeneity of malaria incidence and the seasonality of transmission, which were taken into consideration, are correlated with environmental factors in Mali following a north–south gradient. Indeed, Fig. 4 shows that malaria incidence changes as a function of SEIs, while Figs. 5 and 6 indicate that health districts with the highest incidence of malaria are also those with the highest annual rainfall. These findings, however, must be qualified. Different environmental factors do not have the same impact on malaria transmission. They are also correlated with each other: for instance, a rainstorm simultaneously leads to a decrease in temperature and to an increase in humidity and vegetation growth; a spike in temperature results in a reduction or drying up of water sources; etc. This is supported by our analysis of the relationship between malaria transmission and environmental variables, which found that transmission increases with rainfall, humidity and vegetation growth (positive linear relationship with SEI1) but decreases with temperature (negative non-linear relationship with SEI2). Another issue is that the relationship between malaria transmission and environmental factors varies across and between scales (health districts, regions, country). While our study and the study by Ouedraogo et al.^36^ found a linear relationship between malaria transmission and rainfall/humidity at the national scale, this same relationship was found to be non-linear in Malian studies conducted at the health district scale^16,37^. These observations suggest the importance of accounting for the differentiated and interrelated impact of environmental factors on malaria transmission in future stratifications of malaria risk. Our study can serve as a template for such stratifications in Mali or other malaria endemic countries.

As our findings indicate, 36.4% of the Malian population lives in areas of high malaria transmission, 34.61% in areas of moderate malaria transmission, and 24.3% in areas of low malaria transmission. Only 4.7% of the population lives in areas of very low malaria transmission. However, malaria transmission has evolved considerably over time due to climate change^38^. A study conducted in Dire, a health district of Mali located in the Sahelian zone, found that malaria incidence decreased from 120 to 20 cases per 1000 person-years between 2013 and 2017^37^, suggesting that malaria control interventions also have an impact on the intensity of malaria transmission. Because these factors affect the spatial heterogeneity of malaria incidence, the stratification of malaria risk needs to be updated on a regular basis.

Our study found that the health districts located in the Saharan zone have very low malaria transmission and present few risk factors (Figs. 5 and 6). In these health districts, the main intervention will consist in reinforcing epidemiological surveillance. In the future, mass drug administration and molecular surveillance could also be implemented in these districts^39–41^. Given the resurgence of *Plasmodium vivax* and *Plasmodium malariae* in the Saharan zone^42,43^, diagnosis and treatment could be improved by introducing multi-species RDTs^44,45^. These various strategies could help to achieve malaria pre-elimination. Algeria and Morocco, two countries that border the north of Mali, have succeeded in eliminating malaria using such strategies^46,47^.

In most health districts, the percentage of the population living outside a radius of 5 km from a health facility is very high (Fig. 7). This explains why the malaria diagnosis rate (number of people tested/number of people with malaria symptoms) was only 88% over the period of data collection (2017–2019). To improve the diagnosis and treatment of malaria, iCCM will be implemented in health districts with high child mortality and low health facility usage^48^. Existing iCCM sites will be used and new ones created, and all iCCM sites will be provided with additional diagnostic tools. Since iCCM has shown its efficacy in improving general health and fighting malnutrition in low-income countries like Mali^35,49^, we can expect it to be effective against malaria as well. A study conducted in Zambia has shown that the implementation of iCCM for the diagnosis and treatment of simple malaria improves malaria control^50^.

In both the Saharan zone and the District of Bamako, child mortality is low (under 100 deaths per 1000 population)^2^, as is the prevalence of malaria in children aged 6–59 months (2–10% in the Saharan zone and under 1% in Bamako, Figs. 8 and 9). The low prevalence of malaria in children observed in most West African cities^51^ is explained by urbanization and by the low density of malaria vectors, itself due to low rainfall. In 2018, the NMCP decided to exclude the District of Bamako from SMC interventions and the universal campaign of LLIN distribution because of this low prevalence. However, this decision did not take into consideration variations in malaria transmission levels between urban and peri-urban areas^16^. Peri-urban areas, which are more conducive to malaria transmission, may need to be reintegrated given the higher malaria incidence in Bamako observed in our study.

One of the major interventions selected in our study is the distribution of LLINs, which is recommended by the WHO to further reduce the burden of malaria. A study conducted in Uganda found that LLINs were responsible for a considerable reduction in malaria cases^52^. In Mali, however, insecticide resistance appears to be widespread and could compromise the effectiveness of vector control. To further reduce the parasite load, we initially planned to distribute PBO-LLINs and IG2 nets (instead of standard LLINs) in health districts with high malaria incidence and high vector resistance^13,53^. Yet, because of limited resources and the high cost of these new LLINs, this intervention will be restricted to health districts with proven pyrethroid resistance (25% of all health districts), leaving out neighboring districts with similar climatic characteristics. It should be noted that data on vector resistance are lacking for health districts located in the Saharan zone due to the non-functionality of some sentinel sites (Figs. 10 and 11). The next step will be to map parasite and vector resistance in all health districts to improve the targeting of control interventions.

The IRS intervention was initially planned to be performed in the 11 health districts with the highest malaria incidence. However, due to insufficient financial resources, only 3 health districts located in areas of high transmission will receive IRS in 2022. These 3 districts were selected for two reasons. First, they are located in Mopti region, which has the highest malaria prevalence of the country according to the 2012 DHS. Second, many internally displaced persons (IDPs) have settled in these districts as a result of the security crisis in the north and center of the country. According to the Mali Ministry of Health and a 2021 report by the UN refugee agency, this population alone accounts for 40% of Mali’s IDPs^54^. Given the high vulnerability of IDPs to malaria, this population should also be targeted by high impact interventions.

Malaria case management and IPTp will continue unchanged in all health districts. Free healthcare will be maintained for standard target groups (children aged 6–59 months, pregnant women) and will be provided to new target groups (IDPs).

As the third pillar of the Global Technical Strategy for Malaria 2016–2030, weekly and monthly surveillance of malaria is currently implemented in all health districts regardless of the risk of transmission. This surveillance will be reinforced in the 27 health districts with epidemic potential via the monitoring and training of health care personnel.

A major strength of our study is that the variables used for the selection of interventions are major determinants of transmission in malaria endemic countries, which ensures the reproducibility of our risk stratification. Another strength is that the targeting of interventions takes into consideration the resources that can be mobilized from all actors (government and donors), unlike what is the case in other countries. Indeed, a 2013 review of NMCPs found that most countries target malaria control interventions on the basis of malaria risk maps that use prevalence or incidence data alone^55^. A stratification performed in 2020 in the Harari Region of Ethiopia used only malaria incidence data^56^, while the malaria control interventions implemented in Tanzania in 2020 were based on a stratification of malaria risk that was restricted to incidence data, prevalence data and fever test positivity rates^57^. Compared to these approaches, our strategy allows for a more cost-effective utilization of resources for malaria control.

The main limitation of our study is the insufficiency of data on vector and parasite resistance and prevalence of malaria in children older than 5 years. Indeed, WHO 2017 Malaria Elimination Framework guidelines recommend using such data when conducting malaria risk stratification. Despite this limitation, our work provides an updated risk stratification using advanced statistical methods to inform the targeting of malaria control interventions that can serve as a template for continuous malaria risk stratifications in Mali and in other countries.

To improve the stratification of malaria risk in Mali, the prevalence of malaria at the health district level should be evaluated in future studies. Moreover, studies should be conducted to update the mapping of *Plasmodium* species in areas with very low transmission, particularly in the northern part of the country where an increase in the prevalence of *Plasmodium malariae* and *Plasmodium vivax* has recently been observed^42,43^. Indeed, cases may be missed in measures of malaria transmission due to the fact that current diagnostic tools can only detect *Plasmodium falciparum.*

## Conclusion

In this work, a new stratification of malaria risk using advanced statistical methods was performed to inform the targeting of malaria control interventions in Mali. Eight intervention combinations were discussed and qualitatively selected with the NMCP and the different funding partners for implementation in the country’s different health districts. The targeting of interventions was based on the following variables: spatial heterogeneity of malaria incidence, prevalence of malaria in children aged 6–59 months, distribution of vector resistance, health facility usage, mortality in children aged 6–59 months, and seasonality of transmission. The impact of the intervention on the burden of malaria, the cost of the intervention, and the resources available for the intervention were also taken into consideration. Our work can serve as a template for continuous malaria risk stratifications in Mali and in other countries.

## Supplementary Information

Supplementary Information.

Supplementary Figure S1.

## Data Availability

The data and background maps are available on request from the authors and from the NMCP of Mali.
