# Supplementary material for: Stratification at the health district level for targeting malaria control interventions in Mali
Source: Sci Rep. 2022 May 18;12:8271. doi: 10.1038/s41598-022-11974-3 (PMC9117674; doi:10.1038/s41598-022-11974-3)
Supplement: Supplementary file 1 — Supplementary Information. [file 41598_2022_11974_MOESM1_ESM.docx]

Figure **a and b** show low incidence; Figure **c, d, and e** show high endemicity throughout the year and the seasonality of malaria transmission.; Figure **f** shows a long transmission season with a double peak.
