# Supplementary figures and images for: Stratification at the health district level for targeting malaria control interventions in Mali
Source: Sci Rep. 2022 May 18;12:8271. doi: 10.1038/s41598-022-11974-3 (PMC9117674; doi:10.1038/s41598-022-11974-3)

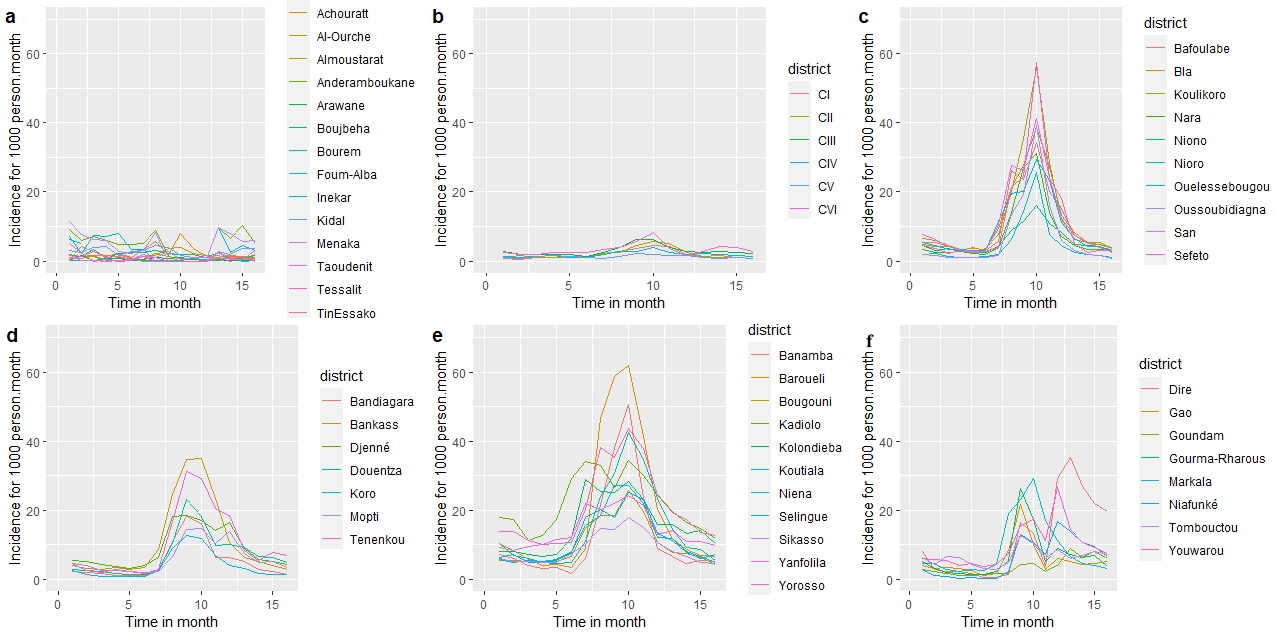

Supplement: Supplementary file 2 — Supplementary Figure S1. [file 41598_2022_11974_MOESM2_ESM.jpeg]
